# Atypical Manifestation of Enterobius vermicularis Infestation in Adults: A Report of a Rare Case

**DOI:** 10.7759/cureus.72074

**Published:** 2024-10-21

**Authors:** Kanya R, Anusha Gopinathan, Shanmuga Leela Arumugam, Han Feliciana J, Leela KV, Balaji D, Harshwanth Chandhar

**Affiliations:** 1 Microbiology, SRM Medical College Hospital and Research Centre, SRM Institute of Science and Technology, Chennai, IND; 2 General Surgery, SRM Medical College Hospital and Research Centre, SRM Institute of Science and Technology, Chennai, IND

**Keywords:** diabetes mellitus, enterobius vermicularis, parasitic disease, pinworm, pruritus ani

## Abstract

*Enterobius vermicularis*, commonly known as pinworm, is a parasitic nematode primarily affecting children, with adult infestations being rare. The infection is typically transmitted via the fecal-oral route and is characterized by intense perianal itching, particularly at night. We present a case of a 39-year-old male with an unusual case of *E. vermicularis* infestation. He initially presented to the outpatient department with complaints of a diabetic foot ulcer, and *E. vermicularis* infestation was an incidental finding. This is an unusual presentation in adults. Adult *Enterobius* infestation must be suspected in patients with comorbidities and poor hygiene who present with persistent perianal pruritus.

## Introduction

*Enterobius vermicularis*, commonly known as pinworm, is a parasitic nematode that primarily infects the human gastrointestinal tract. The worm is named for the distinctive pin-shaped tail found on the posterior end of female worms. It is the most common helminthic infection in developed countries, particularly affecting children between the ages of five and 10 years. *E. vermicularis*, a parasitic worm species from the Oxyurida order, is the causative agent of enterobiasis [1]. The pinworm life cycle begins when eggs are ingested, typically through fecal-oral transmission. The eggs hatch in the small intestine, and the larvae travel to the colon, where they develop into adult worms. At night, adult female pinworms migrate to the perianal area to lay eggs, leading to intense itching, known as pruritus ani or perianal itching. This characteristic symptom often leads to sleep disturbances and secondary bacterial infections due to scratching. While *E. vermicularis* is prevalent in pediatric populations, adult infestations are less common and often underreported. Adults may acquire the infection through close contact with infected children or contaminated environments. Failure to maintain personal and social hygiene leads to the widespread occurrence of the disease and results in significant healthcare expenses for the treatment and management of related complications [2]. Due to the lower prevalence in adults, clinicians may overlook pinworm infection in differential diagnoses of pruritus ani, leading to delays in diagnosis and treatment. The nighttime migration of female worms for egg-laying disturbs sleep, leading to fatigue, difficulty in concentrating, and poor academic performance [3].

This case report presents an unusual instance of *E. vermicularis* infestation in a 39-year-old male, emphasizing the importance of considering parasitic infections in adult patients with persistent perianal itching. The report emphasizes the clinical presentation, diagnosis procedure, treatment, and result, highlighting the necessity for healthcare providers to be cognizant of this condition in adult populations.

## Case presentation

**Figure 1:**
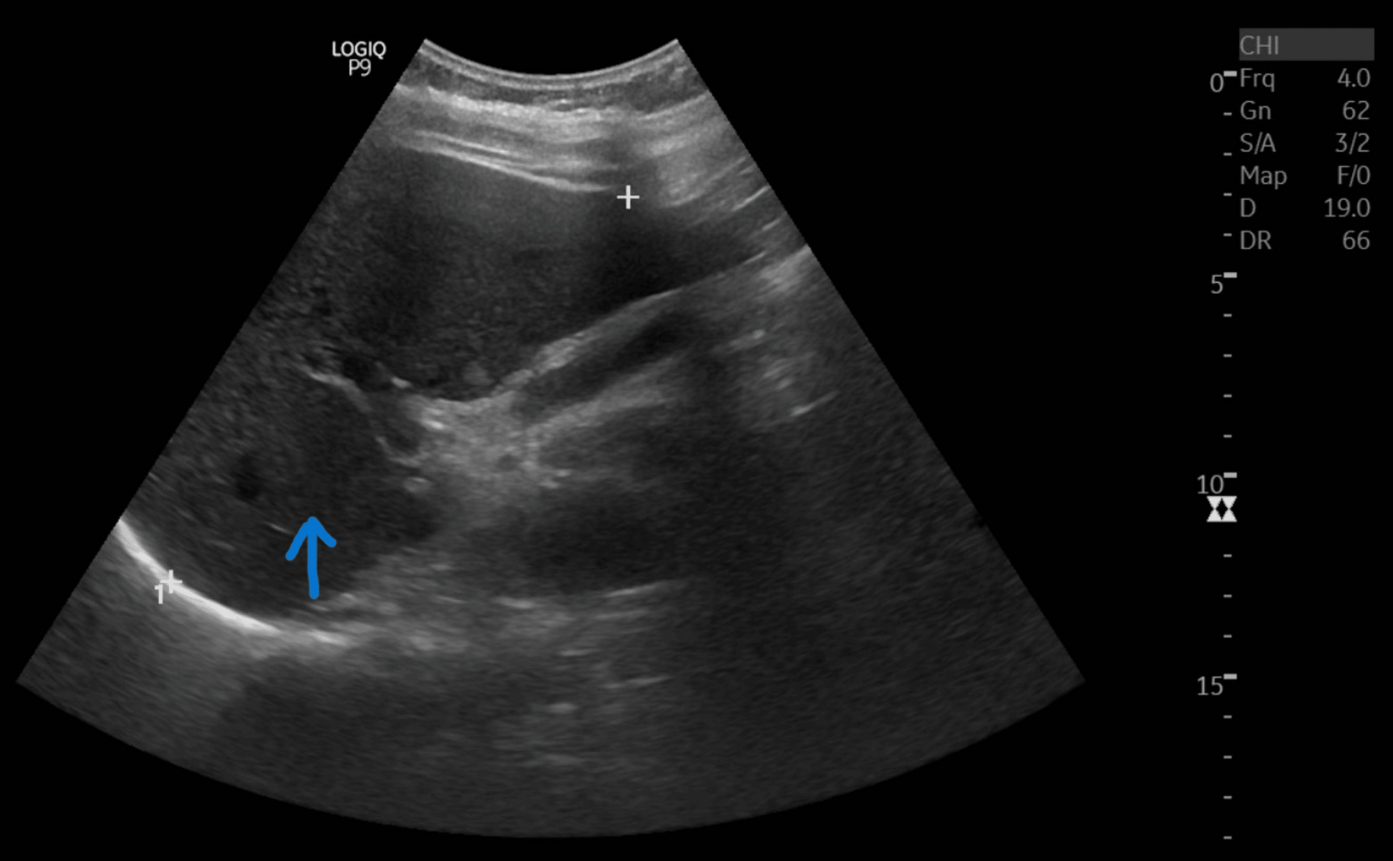
Ultrasound abdomen showing Grade II fatty liver

A 39-year-old male patient from India, diagnosed with type two diabetes mellitus three years ago and receiving irregular treatment for the past 18 months, presented to the outpatient surgery department with complaints of swelling in the right lower limb for the past year and an ulcer present for six months. Initially, he sought treatment at an outside hospital. For the past three days, he noted a foul-smelling discharge from the ulcer site. Additionally, he reported fever with chills and rigors, vomiting, and decreased appetite for the same duration. On general examination, the patient was found to be conscious, oriented, afebrile, and with fair hydration status. No signs of pallor, icterus, clubbing, cyanosis, or lymphadenopathy were observed. His vitals were stable. Systemic examination revealed no significant findings. Local examination of the right lower limb showed swelling extending from the dorsum of the foot up to the knee, accompanied by warmth and tenderness. A 4 x 4-cm ulcer was present over the dorsum of the same foot. An arterial Doppler of the right lower limb revealed mild atheromatous wall calcification in the lower limb arterial system. A right venous Doppler showed no evidence of deep venous thrombosis. An ultrasound of the abdomen revealed Grade II fatty liver (Figure 1).

Wound debridement was performed the next day after admission. The patient was started on piperacillin-tazobactam and metronidazole for seven days along with supportive care and diabetic control based on the institutional antibiogram pattern of wound infections.

**Figure 2:**
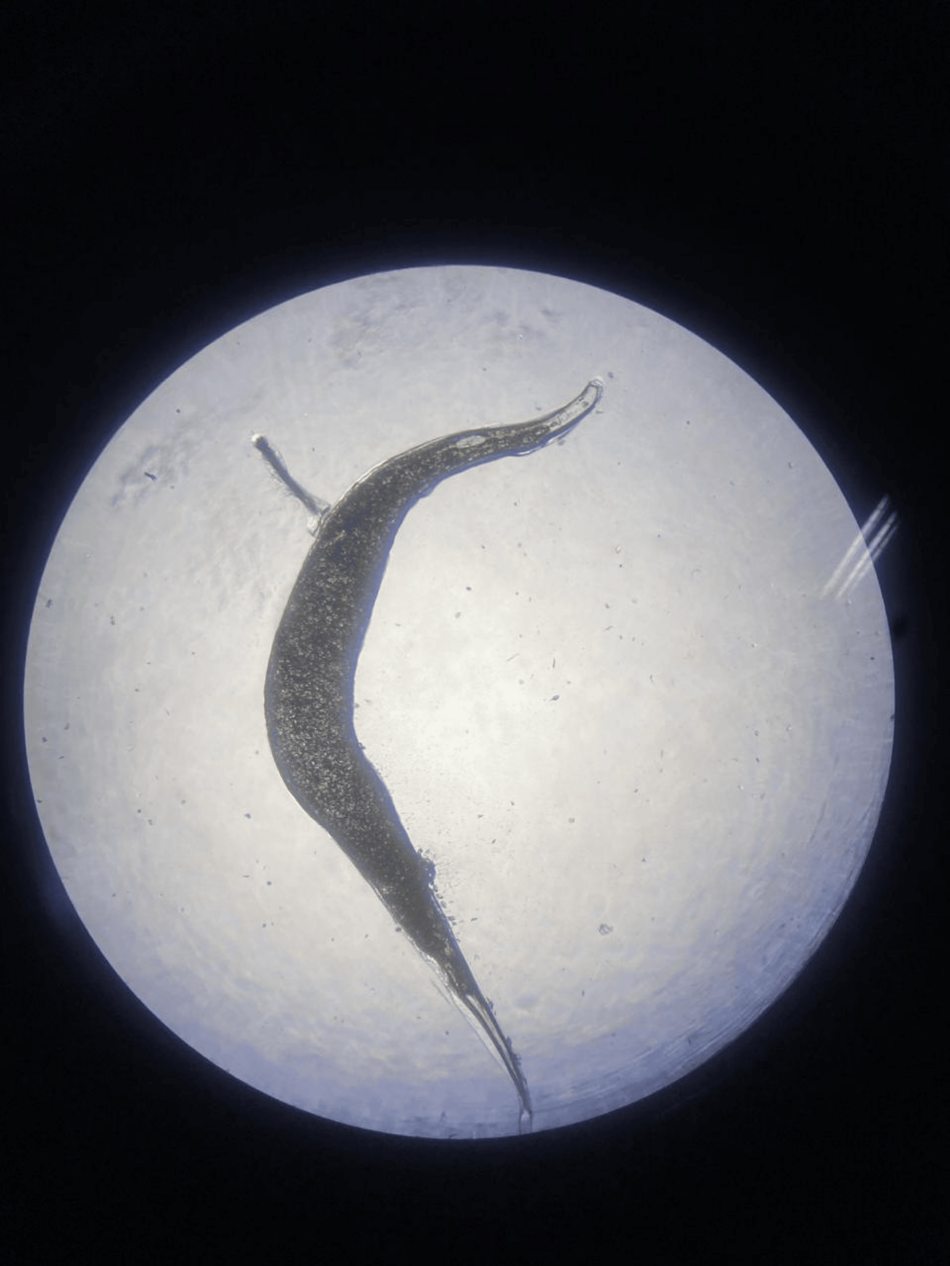
Female pinworm observed in a wet mount preparation of stool sample

**Figure 3:**
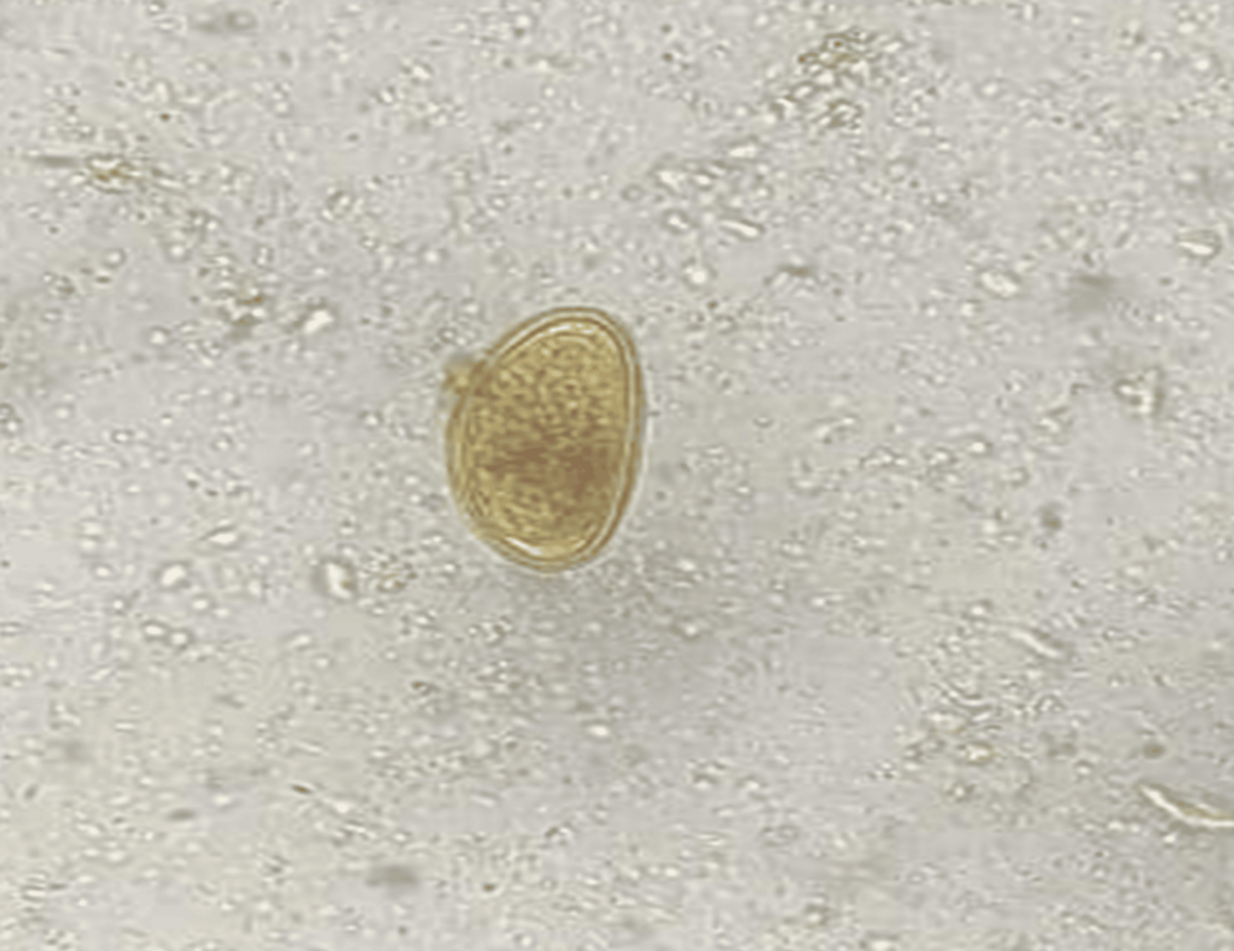
Enterobius vermicularis egg observed in a wet mount preparation of the stool sample

On the second postoperative day, the patient complained of itching in the perianal region. Pus culture and sensitivity of the wound discharge revealed *Enterococcus *spp., susceptible to the beta-lactam group of antibiotics. The foot ulcer started to show signs of improvement. The patient again started complaining of extreme perianal itching with disturbed sleep. Upon further questioning, the patient revealed that he had been suffering from perianal itching for the past six months. Local examination of the perianal region revealed white-colored adult worms in and around the perianal region. The worms were extracted from the perianal region and sent to the microbiology laboratory in a sterile container with saline for identification and further examination. On wet mount preparation of the stool specimen, a female *E. vermicularis* (8 mm x 0.3 mm) (Figure 2) along with eggs of *E. vermicularis* was seen under 40x magnification (Figure 3). 

The eggs (50 µm x 20 µm) were non-bile-stained and asymmetrical and had an elongated oval shape. One side of the egg was slightly flattened while the other side was convex, giving it a D-like appearance, a typical planoconvex shape. A double-layered shell consisting of a smooth outer layer and a granular inner layer was seen. The patient was prescribed tab. Albendazole 400 mg as a stat dose followed by a second dose of the same strength two weeks later to eliminate any possible reinfection. He was also educated about personal hygiene. His family members also received a prophylactic dose of tab. Albendazole 400 mg. The patient improved symptomatically with treatment. The follow-up of the patient was uneventful.

## Discussion

*E. vermicularis*, also known as the human pinworm, is a parasitic nematode that predominantly infects the human gastrointestinal tract. It is estimated that around 200 million people worldwide are affected, with over 30% of cases occurring in children aged five to 10 years [4]. This can be transmitted through the fecal-oral route, fomites, aerosolization, autoinfection, close contact, and environmental contamination [5]. Children living in rural areas with poor sanitation are at a higher risk of contracting helminthic infection than those living in urban areas. The lack of knowledge about proper hygiene practices is a significant factor contributing to the spread of the disease [6]. Humans ingest embryonated eggs from contaminated surfaces or hands, which hatch in the small intestine, releasing larvae migrating to the cecum and appendix, where they mature into adult worms. The adult worms reside in the colon and rectum, where they attach to the mucosa [7]. The presence of worms and their metabolic by-products provoke an immune response, leading to inflammation and irritation. Children, persons living in crowded areas, poor hygiene, lower socio-economic status, environmental contamination, travel, and migration are some of the predisposing factors for pinworm infection [8]. This organism has a direct life cycle that involves human hosts, causing a condition known as enterobiasis. While this infection is generally not severe, it can cause significant discomfort and is highly contagious, particularly among children. The adult *E. vermicularis* exhibits sexual dimorphism, with distinct differences between male and female worms.

The life cycle of *E. vermicularis* is direct and involves only one human host. It begins when embryonated eggs are ingested, typically via contaminated hands, food, or surfaces. Once in the small intestine, the eggs hatch and release larvae that rapidly develop into adult worms. The adult worms then migrate to the colon, where they reside and mate. Females, after mating, migrate to the perianal region, typically at night, to lay thousands of eggs. This nocturnal migration causes intense itching, leading to scratching and subsequent contamination of hands and surroundings with eggs, perpetuating the cycle. Autoinfection happens when an individual scratches the perianal area and inadvertently transfers the infected eggs to their mouth via contaminated hands. The primary habitat for adult *E. vermicularis* worms is the human cecum, appendix, and adjacent areas of the colon. Their presence can cause localized irritation and inflammation of the mucosa. The female’s migration to the perianal region often results in pruritus ani, which can be intensely uncomfortable for the host. The interaction between *E. vermicularis* and its human host is primarily mechanical and irritative. The worms do not invade tissues or cause significant systemic pathology. However, heavy infestations can lead to secondary bacterial infections due to scratching, disturbed sleep, and behavioral issues in affected children.

Diagnosing enterobiasis typically involves identifying eggs or adult worms. The various diagnostic methods available now are the Scotch tape test (sensitivity 90%), National Institute of Health (NIH) swab, microscopic examination of stool samples (sensitivity 5%-15%), serological tests (enzyme-linked immunosorbent assay, immunofluorescence assay, and Western blot), and nucleic acid amplification tests (NAATs) (sensitivity 88.9%, specificity 100%) [9].

Female worms are larger than their male counterparts, typically measuring 8 to 13 mm in length and 0.3 to 0.5 mm in width [10]. They have a slender, fusiform shape that tapers toward both ends. The anterior end features a prominent cephalic expansion, known as the cephalic alae, which is used for attachment to the host’s intestinal mucosa. The posterior end is sharply pointed, giving the worm its common name, "pinworm." Females have a reproductive system that occupies a significant portion of their body cavity. It includes paired ovaries, oviducts, a uterus that can contain thousands of eggs, and a single genital pore located mid-ventrally. The uterus is often seen as a distended structure filled with eggs in mature females [9]. The digestive tract consists of a simple alimentary canal with a mouth, esophagus, intestine, and anus. The esophagus is muscular and cylindrical, leading to a simple tubular intestine that extends the length of the body. The male *E. vermicularis* is smaller, measuring approximately 2 to 5 mm in length and 0.1 to 0.2 mm in width [10]. They are also fusiform but with a more pronounced posterior curvature. Similar to females, males have a cephalic alae at the anterior end. The posterior end features a coiled tail with a single spicule used in copulation. The male reproductive system consists of a single testis, vas deferens, and a copulatory spicule. The spicule is essential for transferring sperm to the female during mating. Like females, males have a straightforward alimentary canal adapted to their parasitic lifestyle.

Eggs are ovoid and asymmetrical and measure about 50 to 60 μm in length. The eggs are usually found in perianal samples obtained using adhesive tape applied to the skin first thing in the morning. They may also be visible in stool samples, though this is less common. The eggs exhibit an ovoid or elongated shape with a unique flattened side, which gives them a slightly asymmetrical appearance, distinguishing them from other helminths. The shell of *E. vermicularis* eggs is thick and composed of two distinct layers. The outer layer is smooth and transparent, providing a clear view of the internal content. The inner layer is more granular in texture. Within the egg, a developing larva is often visible. At the time of egg deposition, the larva is usually at the tadpole stage of development. The presence of this larva is a key identifying feature when viewed under a microscope. The eggs generally appear colorless or may have a slight yellowish tint when observed microscopically. The smooth surface of the egg allows for easy visualization of the internal structures, enhancing the diagnostic process. In fresh specimens, the motile larvae within the eggs can sometimes be observed. Exposure to sunlight and ultraviolet rays can, however, destroy the eggs [2]. These distinct microscopic characteristics of *E. vermicularis* eggs are essential for accurate diagnosis in clinical settings. Antihelminthic agents such as mebendazole, albendazole, pyrantel pamoate, ivermectin, and pyrvinium embonate are used for treatment. Repeat treatment is given after 14 and 28 days to eliminate residual infection. Patients also need to be educated about the modes of transmission and encouraged to perform hand hygiene frequently and to use separate towels and bedsheets. The household contacts of the patient may also be given a single dose of the antihelminthic agent followed by a second dose after 14 days [9].

Intestinal infections in adults are relatively uncommon, with few case reports available, often causing chronic diarrhea or mimicking Crohn’s disease and colitis [11]. Occasionally, pinworms have been found to be masquerading as colorectal or liver masses or causing anaphylaxis after blunt abdominal trauma or even perianal abscess. Many times, the infection is discovered inadvertently during colonoscopy or endoscopy or during the intraoperative period. It occurs in both temperate and tropical climates. Humans are the only natural hosts [4]. The infection ratio of male to female is 2 to 1. However, a higher prevalence of infection is observed in females aged five to 14 years. Previous research has shown that patients with a history of pinworm infection are at a higher risk of developing psychiatric disorders [12]. Additionally, in some cases, enterobiasis is associated with hypereosinophilia [13].

## Conclusions

*E. vermicularis* infection, though typically seen in children, should not be dismissed in adults, especially those with persistent perianal itching and conditions like diabetes, which may increase susceptibility to atypical infections. This case highlights the importance of thorough clinical evaluations, as symptoms can be subtle or misattributed, delaying diagnosis. Clinicians must consider parasitic infections in adults with unexplained symptoms. Early diagnosis and treatment are essential to avoid complications and improve patient outcomes.
